# Chemical Profile, Antioxidant and Cytotoxic Activity of a Phenolic-Rich Fraction from the Leaves of *Brassica fruticulosa* subsp. *fruticulosa* (*Brassicaceae*) Growing Wild in Sicily (Italy)

**DOI:** 10.3390/molecules28052281

**Published:** 2023-03-01

**Authors:** Federica Davì, Maria Fernanda Taviano, Rosaria Acquaviva, Giuseppe Antonio Malfa, Emilia Cavò, Paola Arena, Salvatore Ragusa, Francesco Cacciola, Yassine Oulad El Majdoub, Luigi Mondello, Natalizia Miceli

**Affiliations:** 1 Department of Chemical, Biological, Pharmaceutical and Environmental Sciences, University of Messina, Viale Ferdinando Stagno d’Alcontres 31, 98166 Messina, Italy; 2 Foundation “Prof. Antonio Imbesi”, University of Messina, Piazza Pugliatti 1, 98122 Messina, Italy; 3 Department of Drug and Health Sciences, University of Catania, Viale A. Doria 6, 95125 Catania, Italy; 4 Research Centre on Nutraceuticals and Health Products (CERNUT), University of Catania, Viale A. Doria 6, 95125 Catania, Italy; 5 PLANTA/Autonomous Center for Research, Documentation and Training, Via Serraglio Vecchio 28, 90123 Palermo, Italy; 6 Department of Biomedical, Dental, Morphological and Functional Imaging Sciences, University of Messina, Via Consolare Valeria, 98125 Messina, Italy; 7 Chromaleont s.r.l., c/o Department of Chemical, Biological, Pharmaceutical and Environmental Sciences, University of Messina, Viale Palatucci, 98168 Messina, Italy; 8 Department of Sciences and Technologies for Human and Environment, University Campus Bio-Medico of Rome, Via Àlvaro del Portillo 21, 00128 Rome, Italy

**Keywords:** Mediterranean cabbage, Sicilian vascular flora, edible wild plants, traditional medicine, polyphenols, biological activities

## Abstract

Recently, our research team has started a study on *Brassica fruticulosa* subsp. *fruticulosa*, an edible plant traditionally used to treat various ailments, little investigated to date. Good in vitro antioxidant properties were highlighted for the leaf hydroalcoholic extract, with the secondary higher than the primary ones. In continuation of the ongoing research, this work was designed to elucidate the antioxidant properties of the phenolic compounds contained in the extract. For this purpose, a phenolic-rich ethyl acetate fraction (*Bff*-EAF) was obtained from the crude extract by liquid–liquid extraction. The phenolic composition was characterized by HPLC-PDA/ESI-MS analysis and the antioxidant potential was investigated by different in vitro methods. Furthermore, the cytotoxic properties were evaluated by MTT, LDH and ROS determinations on human colorectal epithelial adenocarcinoma cells (CaCo-2) and human normal fibroblasts (HFF-1). Twenty phenolic compounds (flavonoid and phenolic acid derivatives) were identified in *Bff*-EAF. The fraction exhibited good radical scavenging activity in the DPPH test (IC_50_ = 0.81 ± 0.02 mg/mL), and moderate reducing power (ASE/mL = 13.10 ± 0.94) and chelating properties (IC_50_ = 2.27 ± 0.18 mg/mL), contrary to what previously observed for the crude extract. *Bff*-EAF reduced in a dose-dependent manner CaCo-2 cell proliferation after 72 h of treatment. This effect was accompanied by the destabilization of the cellular redox state due to the antioxidant and pro-oxidant activities displayed by the fraction at lower and higher concentrations. No cytotoxic effect was observed on HFF-1 fibroblasts, used as control cell line.

## 1. Introduction

Phenolic compounds have been reported to exhibit beneficial biological effects on human health; indeed, many epidemiological research data strongly suggest that long-term consumption of polyphenol-rich plant products plays an important part in the prevention of chronic diseases such as cancer, cardiovascular disease, obesity, and neurodegenerative diseases [1,2].

In recent years, several taxa included in the *Brassicaceae* family have been a fascinating research topic, as they represent a rich mine of bioactive phytochemicals with beneficial effects on health improvement. Within this family, *Brassica* L. is one of the most economically important genera, including several species widely cultivated throughout the world [3]. The health-promoting effects of *Brassica* species have been partly attributed to their antioxidant capacity, mainly related to the presence of phenolic compounds, which represent the major group of phytochemicals with antioxidant ability [4,5,6,7]. The most widespread phenolics in *Brassica* species are flavonoids (mainly flavonols, but also anthocyanins) and hydroxycinnamic acids, whose qualitative and quantitative profiles vary significantly among species. Quercetin, kaempferol, isorhamnetin are the main representative flavonols in these species, which can be found free or conjugated with sugars or acylated with different hydroxycinnamic acids. The most common hydroxycinnamic acids are ferulic acid, sinapic acid, caffeic acid, and p-coumaric acid, differently conjugated to organic acids and sugars [5,6,8,9,10].

Previous studies have documented the nutritional and medicinal properties of the edible wild *Brassica* species respect to cultivated crops, indicating their great potential as sources of bioactive compounds [8,11,12]. *Brassica fruticulosa* Cyr. subsp. *fruticulosa* (Mediterranean cabbage) is an herbaceous species, generally biennial to perennial, with Mediterranean distribution; this species is widespread in Southern Italy, including Sicily [13]. The use of *B. fruticulosa* subsp. *fruticulosa* in traditional Sicilian medicine has been reported by some authors as an antidiabetic, in the treatment of gastric and respiratory tract diseases, to treat superficial wounds and raise blood pressure [14,15,16]. In addition, digestive properties are attributed to this species, but also mineralizing, due to the high content of mineral salts, and tonic properties, due to the high content of vitamins A and C [14]. *Brassica fruticulosa* subsp. *fruticulosa* is an edible plant, used since ancient times for food purposes both raw and cooked; indeed, this species was widely utilized in the past as an important survival food from the poorest populations [14]. In southern Italy, especially in Sicily, young shoots and leaves are utilized to prepare typical dishes [17,18,19].

Despite its use in folk medicine and as an edible plant, *B. fruticulosa* subsp. *fruticulosa* has been very little studied to date; in fact, in the literature, only a few studies can be found concerning the chemical characterization, while no research on the biological properties has been previously reported [13]. To deepen the knowledge of this species, a study has recently been conducted by our research team aimed at evaluating the bioactive potential of a hydroalcoholic extract obtained from the leaves of *B. fruticulosa* subsp. *fruticulosa* that grows spontaneously in Sicily [13]. This experimental study has been carried out within a larger project planned for the valorization of underutilized species belonging to the genus *Brassica* growing wild in Sicily, through the chemical and biological characterization of the active secondary metabolites. The investigations led to the identification of several phenolic compounds, highlighting the antioxidant properties of *B. fruticulosa* subsp. *fruticulosa* leaf extract; particularly, radical scavenging and ferrous ion chelating activities were observed using in vitro tests based on different mechanisms, the latter being greater than the former, whereas only mild reducing power was evidenced. Phenolic compounds are known to act as antioxidants through several chemical mechanisms, directly as reducing agents or hydrogen donors, but also by indirect mode of action as metal-chelators; thus, it was hypothesized that the antioxidant properties of the extract could be related to some extent to these compounds [13]. As a continuation of the ongoing research, the current work was designed with the aim of defining the antioxidant ability of phenolic compounds contained in the *B. fruticulosa* subsp. *fruticulosa* leaf extract. For this purpose, a phenolic-rich ethyl acetate fraction (*Bff*-EAF) was obtained from the crude extract by liquid–liquid extraction. The profile of flavonoids and phenolic acids contained in the fraction was characterized by HPLC-PDA/ESI-MS analysis and the antioxidant potential was investigated by the same in vitro methods previously utilized for the crude extract, in order to elucidate the mechanisms involved in the antioxidant effectiveness of these phenolic compounds. Furthermore, the cytotoxic properties of the fraction were evaluated on human colorectal epithelial adenocarcinoma cells (CaCo-2) and human normal foreskin fibroblasts (HFF-1); the levels of ROS in cells were also determined, in order to establish whether the cytotoxic effect of the fraction could be related to the antioxidant or pro-oxidant action of the phenolic compounds. 

## 2. Results

### 2.1. Phytochemical Investigations

#### 2.1.1. Determination of Total Phenolic Content

The Folin–Ciocâlteu test is a colorimetric method based on electron transfer reactions between the Folin–Ciocâlteu reagent and phenolic compounds in alkaline conditions, giving rise to the formation of a blue chromophore with the maximum absorption at 765 nm. Generally, gallic acid is used as the reference standard compound, and the results are expressed as gallic acid equivalent [20]. The procedure is widely used for quantification of total phenolic compounds in plant extracts; indeed, in most cases, the antioxidant properties of plant extracts are explained by their total phenolic content with good correlation, confirming the value of the test.

The results of this colorimetric method highlighted that the total phenolic content in *Bff*-EAF was equal to 107.44 ± 1.85 mg gallic acid equivalent (GAE)/g fraction. 

#### 2.1.2. Identification of Phenolic Compounds by HPLC-PDA/ESI-MS

**Figure 1:**
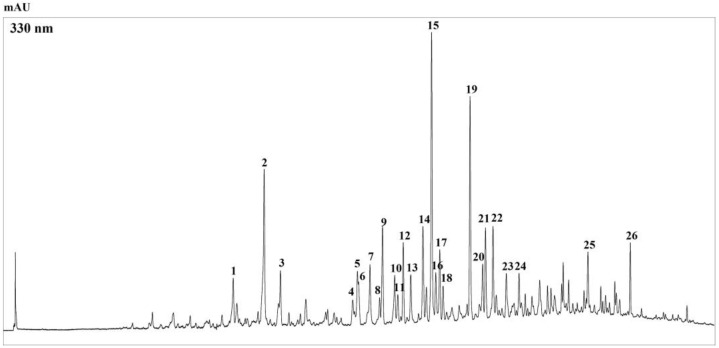
HPLC-PDA chromatograms of the polyphenolic compounds, extracted at 330 nm wavelenght, of *Bff*-EAF. For peak identification, see Table 1.

The HPLC-PDA chromatogram (ʎ = 330 nm) of the phenolic compounds present in *Bff*-EAF is shown in Figure 1. The analysis led to the separation of 26 compounds; through the complementary information provided by the retention times and by the PDA, MS and MS/MS spectra, further supported by data reported in the literature, a total of 20 compounds were identified, 11 belonging to the flavonoid class and 9 to that of phenolic acids. Table 1 shows the peaks detected, with the retention times, the [M-H]^-^ values, and the characteristic UV/Vis maxima; furthermore, when observed, the secondary fragments of the ions resulting from further fragmentation of the precursor ion [M-H]^-^ were also reported.

The flavonoids detected in the fraction are derivatives of the flavonols: kaempferol, quercetin and isorhamnetin. As for phenolic acids, they are derivatives of the hydroxycinnamic acids: sinapic acid and ferulic acid.

To achieve the quantification of flavonoids, since none of the compounds identified were commercially available, three reference standards were considered, namely, quercetin-3-*O*-glucoside, kaempferol-3-*O*-glucoside, and isorhamnetin-3-*O*-glucoside, for the determination of quercetin, kaempferol, and isorhamnetin derivatives, respectively. The quantitative determination was carried out by interpolation of the calibration curves obtained with the standards. As shown in Table 1, among the flavonoids identified the derivatives of kaempferol represent the major compounds; kaempferol-3-triglucoside (peak n. 15) was the main component, followed by peak n. 19, kaempferol-feruloyldihexoside. Phenolic acid derivatives have not been quantified.

### 2.2. Antioxidant Activity

**Figure 2:**
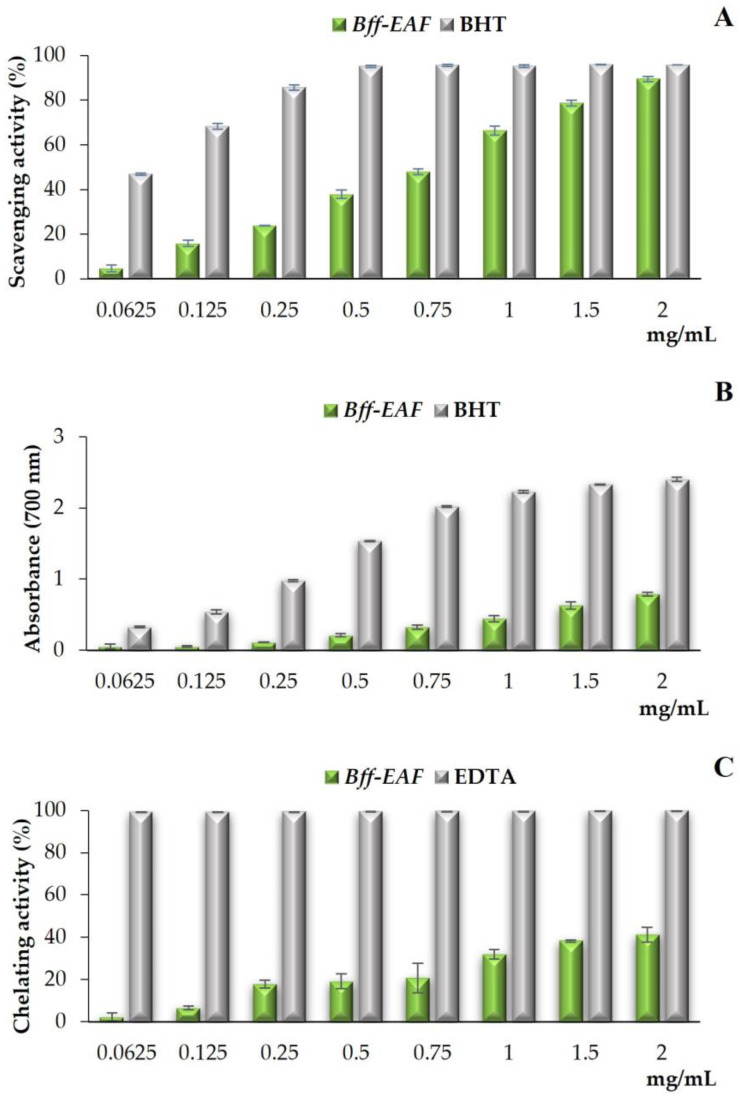
Free radical scavenging activity (DPPH test) (**A**), reducing power (**B**), and ferrous ion chelating activity (**C**) of *Bff*-EAF. Values are expressed as the mean ± SD (*n* = 3).

DPPH (2,2-diphenyl-1-picrilidrazil) is a stable radical characterized by the delocalization of the spare electron; the methanolic solution of DPPH has a purple color, which turns yellow when the radical accepts a proton from a “scavenger” antioxidant with the formation of the reduced form DPPH-H (2,2-diphenyl-1-picrylhydrazine). In its radical form, DPPH absorbs at 517 nm, but following its reduction by an antioxidant agent (AH) the absorption disappears. The degree of discoloration of the solution indicates the amount of reduced DPPH; the greater the discoloration, the higher the antioxidant activity [25]. The results of the DPPH test showed that *Bff*-EAF displayed good free radical scavenging activity, which increases with increasing concentration (Figure 2A). At the highest concentration tested, the activity of the fraction was approximately 90%, close to that of the BHT standard (about 95%). The IC_50_ values calculated for the fraction and the standard were found to be 0.81 ± 0.02 mg/mL and 0.07 ± 0.01 mg/mL, respectively.

The reducing power of the fraction was assessed by evaluating the ability to reduce the trivalent iron of ferricyanide to divalent iron and measuring the intensity of the green-blue color obtained. The intensity of the color is directly related to the concentration of Fe^2+^ ions, which in turn depends on the reducing capacity of the sample. On the basis of the reducing power, the color changes from the initial yellow to a shade between green and blue; a higher absorbance value corresponds to a higher reducing power [26]. Figure 2B shows the results of the reducing power evaluated for *Bff*-EAF; the fraction exhibited moderate activity, as compared with the standard BHT, which increases with increasing concentration. The absorbance values detected for the fraction, in fact, reach approximately 0.8 at the highest tested concentration, about one third compared to that of the BHT standard, resulting about 2.4. The calculated ASE/mL values were 13.10 ± 0.94 and 0.89 ± 0.06, for the fraction and the standard, respectively.

To estimate the chelating activity of the fraction, the formation of the Fe^2+^-ferrozine complex was measured. Ferrozine (3-(2-pyridyl)-5,6-diphenyl-1,2,4-triazine-4′,4″-disulfonic acid sodium salt) forms red complexes with divalent iron; in the presence of chelating agents, there is a stabilization of the ferrous ions, with consequent reduction of the formation of the complex Fe^2+^-ferrozine and, therefore, of the red color of the solution. The measurement of the reduction in color intensity, therefore, allows us to estimate the chelating activity of the extract [26]. The results of the ferrous ion chelating activity assay are shown in Figure 2C; from the comparison with the EDTA standard, it is evident that *Bff*-EAF has moderate and concentration-dependent chelating properties, reaching approximately 40% at the highest tested concentration. This was confirmed also from the IC_50_ values calculated for the fraction and the standard, which were 2.27 ± 0.18 mg/mL and 0.007 ± 0.001 mg/mL, respectively.

### 2.3. Cytotoxic Activity

#### 2.3.1. Cell Viability

**Figure 3:**
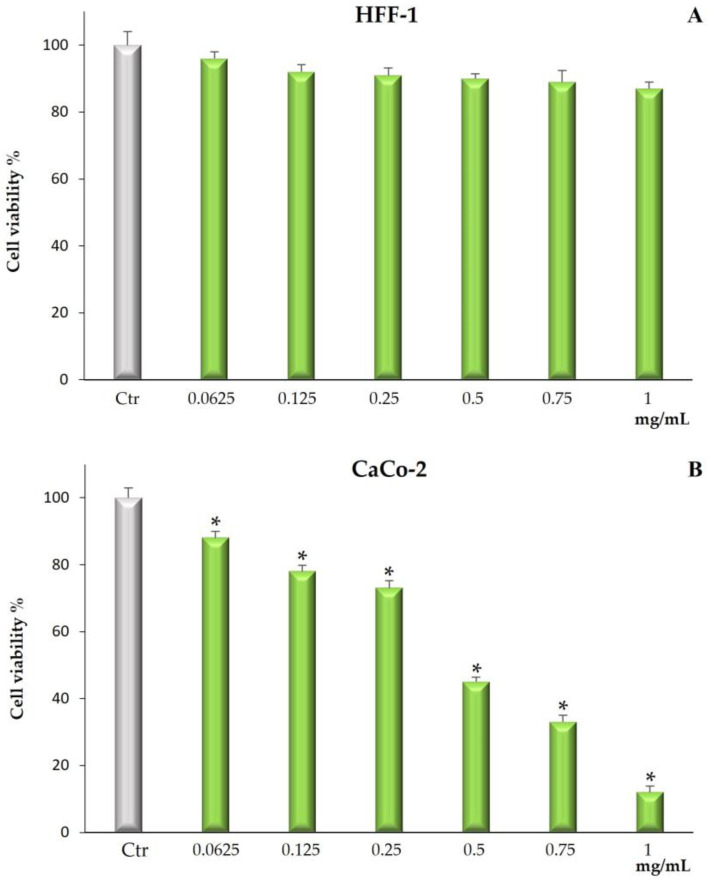
Cell viability in HFF-1 (**A**) and CaCo-2 (**B**) cells untreated and treated for 72 h with *Bff*-EAF at different concentrations (0.0625–1 mg/mL). Values are the mean ± SD of four experiments in triplicate. * Significant vs. untreated control cells: *p* < 0.001.

Cytotoxicity was determined by measuring cell viability by MTT assay. In human normal fibroblast HFF-1 cells, used as control cell line, *Bff*-EAF did not exert any toxic effect at all tested concentrations for 72 h (Figure 3A). The fraction, on the other hand, exerted a significant cytotoxic effect on the CaCo-2 cell line starting from the lowest concentration (0.0625 mg/mL). Figure 3B shows, in fact, that the treatment for 72 h was able to reduce, in a dose dependent manner, cell viability at all tested concentrations (0.0625–1 mg/mL).

#### 2.3.2. LDH Release

**Figure 4:**
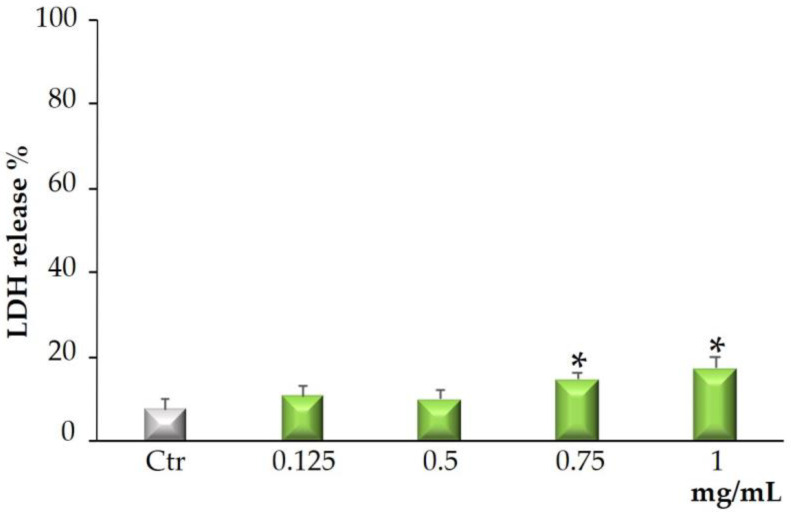
LDH released in CaCo-2 cells untreated and treated for 72 h with *Bff*-EAF at different concentrations (0.125–1 mg/mL). Values are the mean ± SD of four experiments in triplicate. * Significant vs. untreated control cells: *p* < 0.001.

Figure 4 shows that treatment with *Bff*-EAF is unable to induce the release of LDH at 0.125–0.5 mg/mL, while at the concentrations of 0.75 and 1 mg/mL the LDH release was increased significantly with respect to the control cells. The obtained results in the LDH assay confirmed that most of the antiproliferative activity of the fraction, observed by the MTT assay, is associated with necrotic cellular death only at the higher concentrations.

#### 2.3.3. Reactive Oxygen Species (ROS)

**Figure 5:**
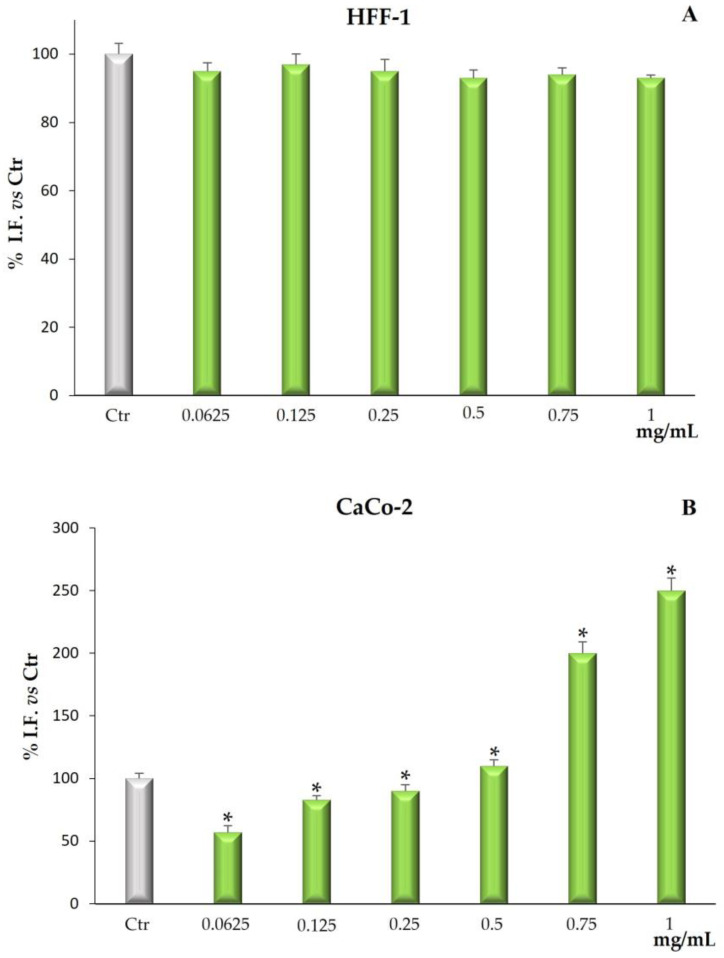
ROS levels in HFF-1 (**A**) and CaCo-2 (**B**) cells untreated and treated for 72 h with *Bff*-EAF at different concentrations (0.0625–1 mg/mL). Values are the mean ± SD of four experiments in triplicate. * Significant vs. untreated control cells: *p* < 0.001.

Alteration of redox homeostasis in cancer cells may promote the expression of multiple proteins affecting cell death rather than survival. Increased levels of ROS can lead to the activation of cell death processes such as apoptosis and ferroptosis [27]. Therefore, we investigated whether *Bff*-EAF-induced cell death correlated with an alteration of ROS levels. As depicted in Figure 5A, in normal fibroblasts HFF-1, used as control cells, no variation in radical species levels was observed at all fraction concentrations tested. Otherwise, in CaCo-2 cells *Bff*-EAF induced a significant ROS level decrease at the lowest concentration of 0.0625 mg/mL. At the concentrations of 0.125–0.250 mg/mL, it caused a slightly significative dose-dependent increase in ROS levels near the values of untreated cells. Surprisingly, starting from the dose of 0.5 mg/mL, *Bff*-EAF increased ROS levels causing a severe oxidative stress condition at the highest concentrations tested (Figure 5B).

## 3. Discussion

Recently, our research group designed a study aimed at evaluating the bioactive potential of a hydroalcoholic extract obtained from the leaves of *B. fruticulosa* subsp. *fruticulosa* growing wild in Sicily (Italy), never investigated before. The research previously carried out has led to the characterization of numerous phenolic compounds, highlighting the in vitro antioxidant properties of the extract [13].

Antioxidants have been classified into two main types, based on their mechanism of action: the primary antioxidants (chain breaking) react with free radicals producing less reactive species or disrupting the chain propagation processes; the secondary antioxidants indirectly inhibit oxidation through various mechanisms, including the chelation of metal ions [28,29]. It is well known that phenolic compounds, including phenols, phenolic acids, flavonoids, tannins and lignans, represent the main class of natural antioxidants. They act as antioxidants in several ways: as “radical scavengers” due to the presence of phenolic groups which are excellent nucleophiles; as lipid peroxidation inhibitors, interrupting the oxidation reaction by binding to free radicals. In addition, phenolic compounds act as chelating agents of metal ions that induce oxidation [30,31].

Based on these assumptions and in the continuation of the research undertaken, the antioxidant potential of phenolic compounds contained in the leaf hydroalcoholic extract of *B. fruticulosa* subsp. *fruticulosa* was investigated in the present study. For this purpose, the extract was subjected to fractionation by solvent partitioning method and subsequently the ethyl acetate fraction (*Bff*-EAF) was selected as phenolic-rich fraction to be used for our investigations, as confirmed through the determination of the total phenolic content by the Folin–Ciocâlteu assay. The characterization of the phenolic compounds contained in *Bff*-EAF was attained by HPLC-PDA/ESI-MS analysis, which led to the identification of flavonoids, represented by kaempferol, quercetin and isorhamnetin derivates, and phenolic acids, sinapic and ferulic acid derivates. 

It is well known that the antioxidant activity of a phytocomplex cannot be estimated by a single method, due to the complex nature of its constituents and their interactions; in order to take into account the various mechanisms by which plant-based phytochemicals act as antioxidants in vivo, it is, therefore, necessary to perform more than one type of antioxidant capacity measurement based on a different mechanism of action [6,32]. Thus, the antioxidant potential of *Bff*-EAF was assessed through three different in vitro methods: the primary antioxidant properties were investigated using the DPPH test, an electron/hydrogen atom transfer (ET/HAT, mixed mode) based assay, and reducing power assay, based on the ET mechanism; the secondary antioxidant properties were determined by testing the chelating activity of ferrous ions [13,28]. The phenolic-rich fraction *Bff*-EAF showed good primary antioxidant properties, which appear to be mainly based on a hydrogen atom transfer mechanism. Indeed, in the DPPH test, good radical scavenging activity was highlighted for the fraction, close to that of the standard BHT at the highest tested concentration (2 mg/mL), whereas only a moderate reducing power was observed. On the other hand, based on the results of the ferrous ion chelating activity assay, *Bff*-EAF was found to possess moderate secondary properties, reaching approximately 40% inhibition of the ferrozine-Fe^2+^ complex formation at the highest tested concentration. 

Contrary to the fraction, the crude extract of *B. fruticulosa* subsp. *fruticulosa*, previously tested through the same in vitro experimental methods, had shown good secondary antioxidant properties, higher than the primary ones [13]. Therefore, based on the results obtained in this study, it is possible to state that the phenolic compounds contained in the fraction are mainly responsible for the primary antioxidant properties highlighted in the crude extract, and hydrogen atom transfer seems to be the main mechanism involved; instead, it appears evident that the good chelating activity of the *B. fruticulosa* subsp. *fruticulosa* leaf extract can only partially be traced back to these compounds.

Current scientific knowledge attributes to species belonging to *Brassica* genus several health benefits such as reduced risk of cancer [33,34]. Numerous studies have demonstrated the ability of polyphenols to modulate oxidative stress in tumor cells, thus affecting signal transduction, the activation of redox-sensitive transcription factors and the expression of specific genes affecting cell proliferation and apoptosis. On the other hand, pro-oxidant effects of polyphenols have also been described; indeed, polyphenols can cause the formation of ROS to reach an unbearable level of oxidative stress in cancer cells, which induces apoptosis and cell death, and blocks cell proliferation. Therefore, polyphenols can neutralize the constitutive ROS or generate additional quantities of ROS, depending on the concentration and the cellular environment, causing, in both cases, the inhibition of tumor cell proliferation [35,36]. 

Given the good in vitro antioxidant properties highlighted for *Bff*-EAF, it seemed interesting to evaluate the cytotoxic properties of this phenolic-rich fraction on a human colorectal epithelial adenocarcinoma cell line (CaCo-2). The results of the MTT test showed a significant cytotoxic activity of the fraction after cell exposure for 72 h at all the concentrations assayed; conversely, no cytotoxic effect was observed on the normal HFF-1 fibroblasts, used as control cell line. Previous studies by this research group proved that extracts from *Brassica incana* Ten. grown wild in Sicily (Italy), also containing derivatives of the flavonols quercetin, kaempferol and isorhamnetin, and of the hydroxycinnamic acids sinapic acid and ferulic acid, were able to reduce cell viability in the same tumor cell line [23]. It is well documented that the high amount of phytochemicals contained in a vegetable-based diet effectively reduces the risk of digestive tract cancers, including colorectal cancer [37]. In our results *Bff*-EAF, at the concentrations tested in the range 0.0625 to 1 mg/mL, reduced CaCo-2 cell proliferation in a dose-dependent manner. The reduced viability induced by the fraction is not strictly linked with necrotic cell death, which is appreciable only at the highest concentrations (0.750 and 1 mg/mL) but significantly lower than the MTT values, therefore not correlatable between them. The above results could be attributable to a different mechanism of action probably related to the modulation of ROS levels and the destabilization of redox state that can lead to the activation of cell death processes such as apoptosis [38,39]. Our results confirm that natural substances can play both antioxidant and pro-oxidant roles. In addition, it is known that most of the natural antioxidants in the diet can act as both free radical scavengers and metal chelators. Thanks to these properties, the presence of natural antioxidants in the diet could help counteract the toxic effects of ROS [38]. However, some natural compounds, such as polyphenols, can behave as pro-oxidants and this can generate oxidative damage by inducing cytotoxicity in cancer cells [39]. The results of our research suggest that the phenolic-rich fraction *Bff*-EAF can exert its activity by destabilizing the cellular redox balance, also involving intracellular target factors of ROS. Surely ROS production is a key factor contributing to carcinogenesis. In a neoplastic cell, persistent oxidative stress may be responsible for activating growth factor pathways, increasing resistance to apoptosis and inducing cell death through a process called ferroptosis [40]. Further research should be conducted to better elucidate additional mechanisms responsible for the observed cytotoxic effect (apoptotic and or ferroptotic death) and the metabolic pathways involved in the transduction of intracellular signals.

## 4. Materials and Methods

### 4.1. Chemicals and Reagents

LC–MS-grade water (H_2_O), acetonitrile (ACN), isorhamnetin-3-*O*-glucoside, quercetin-3-*O*-glucoside, kaempferol-3-*O*-glucoside, Folin–Ciocâlteu reagent, sodium carbonate, gallic acid, 2,2-diphenyl-1-picrylhydrazyl, butylated hydroxytoluene (BHT), potassium hexacyanoferrate (III), iron (III) chloride hexahydrate, L-ascorbic acid, sodium phosphate monobasic monohydrate, potassium phosphate dibasic, trichloroacetic acid, iron (II) chloride, 3-(2-Pyridyl)-5,6-diphenyl-1,2,4-triazine-4′,4″-disulfonic acid sodium salt, ethylenediaminetetraacetic acid (EDTA), CaCo-2 (human colorectal adenocarcinoma, ATCC^®^, HTB-37)—ATCC, Rockville, MD, USA, HFF-1 (Human Foreskin Fibroblasts, ATCC^®^ SCRC-1041.1)—ATCC, Rockville, MD, USA, Minimum Essential Medium Eagle (MEM), Dulbecco’s Modified Eagle Medium (DMEM), fetal bovine serum (FBS), penicillin/streptomycin, tetrazolium salts, β-nicotinamide-adenine dinucleotide (NADH), phosphate buffered saline (PBS), 2′,7′-dichlorofluorescein diacetate (DCFH-DA), and digitonin were obtained from Merck Life Science (Merck KGaA, Darmstadt, Germany). LC–MS-grade formic acid was purchased from Riedel-de Haën (Seelze, Germany). Methanol (MeOH) was purchased from Carlo Erba (Milan, Italy).

### 4.2. Plant Material and Extraction Procedure

The leaves of *Brassica fruticulosa* subsp. *fruticulosa* were harvested in October 2019 in the locality of Massa San Giorgio, in the Peloritani Mountains (Messina, Sicily, Italy). The taxonomic identification was confirmed by Prof. S. Ragusa, Department of Health Sciences, University “Magna Graecia” of Catanzaro (Catanzaro, Italy). A voucher specimen (1016/19) was deposited in the same Department.

After collection, the plant material was washed, blended, frozen, and then lyophilized. The *B. fruticulosa* subsp. *fruticulosa* leaf hydroalcoholic extract was obtained by the procedure reported in our previous work [13]. After preventive maceration of finely ground leaves at 25 °C for 1 h, the extraction was performed with 70% MeOH (1:10 *w*/*v*) in an ultrasonic bath at 50 °C for 15 min. The extraction procedure was repeated three times; then, the filtrates were combined and evaporated to dryness by a rotavapor. The crude extract obtained was then subjected to fractionation using the liquid–liquid extraction method in a separating funnel. The crude extract was suspended in distilled water (1:10 *w*/*v*) and subsequently fractionated by partitioning with organic solvents of increasing polarity (1:1): *n*-hexane, dichloromethane and ethyl acetate; then, the ethyl acetate phases were pooled and evaporated to dryness by rotavapor. The yield of the ethyl acetate fraction (*Bff*-EAF) selected for the present study, referred to 100 g of crude extract, was equal to 2.66%.

### 4.3. Phytochemical Investigations

#### 4.3.1. Determination of Total Phenolic Content

The total phenolic content of *Bff*-EAF was measured by using by the Folin–Ciocâlteu colorimetric assay, referring to calibration curve of gallic acid, according to the method reported by Gao et al. [41]. An aliquot of 0.1 mL of each sample solution was mixed with 0.2 mL Folin–Ciocâlteu reagent, 2 mL of distilled water, and 1 mL of 15% *w/v* sodium carbonate. The absorbance was measured at λ = 765 nm, after a 2 h incubation in the dark at room temperature, with an UV–Vis spectrophotometer (model UV-1601, Shimadzu, Milan, Italy).

The results were obtained from the average of three independent determinations and are expressed as mg gallic acid equivalents (GAE)/g fraction (dw) ± standard deviation (SD).

#### 4.3.2. Identification of Phenolic Compounds by HPLC-PDA/ESI-MS

A quali-quantitative investigation of the phenolic compounds contained in *Bff*-EAF has been carried out using a Shimadzu HPLC system (Milan, Italy) equipped with a CBM-20A controller, two LC-20AD pumps, a DGU 20A3 degassing system, a SIL-20AC autosampler, an SPD-M20A photo diode array detector (PDA), and a triple-quadrupole mass analyzer (LCMS-8050, Shimadzu, Kyoto, Japan), equipped with an ESI interface, in positive and negative ionization mode [42,43,44]. Data acquisition was performed by Shimadzu LabSolution software ver. 5.91.

*Sample preparation:* For the analysis, 1.1 mg of the fraction was dissolved in 1 mL of MeOH.

*Chromatographic conditions:* Analyses were carried out using an Ascentis Express C18, 15 cm × 4.6 mm internal diameter (i.d.), with particle size of 2.7 µm (Merck Life Science, Merck KGaA, Darmstadt, Germany). The injection volume was 20 µL, and the mobile phase consisted of water/formic acid (99.9:0.1, *v*/*v*) (solvent A) and ACN/formic acid (99.9:0.1, *v*/*v*) (solvent B); the linear gradient profile was the following: 0–15 min, 0–5% B; 15–65 min, 20% B; 65–95 min, 35% B; 120 min, 100% B. The flow rate for separation and detection was 1 mL/min, and it was split to 0.2 mL/min prior to MS detection.

*PDA conditions:* The wavelength range was 200–400 nm, and the chromatograms were extracted at 330 nm. The time constant was 0.08 s, and the sample frequency was 40 Hz.

*MS conditions:* The MS acquisition was performed using the ESI interface in negative ionization mode. Mass determination was performed in full scan mode under the following conditions: mass spectral range: 100–1000 *m*/*z*; interval: 0.5 s; nebulizing gas flow (N_2_): 3 L/min. The instrument has been set as follows: interface temperature: 300 °C; heat block: 400 °C; DL temperature: 250 °C; DL voltage: −34 V; probe voltage: 4.5 kV; Q-array voltage: 1.0 V; RF voltage: 90 V; detection gain: 1.0 kV.

*Quantitative determination:* For the semi-quantification of the identified polyphenols, calibration curves of three standards representative of the chemical classes under study were used, namely, isorhamnetin-3-*O*-glucoside (*y* = 14948*x* − 2966.9; limit of detection (LOD) = 0.032, limit of quantification (LOQ) = 0.098), quercetin-3-*O*-glucoside (*y* = 13424*x* + 898.59; LOD = 0.013, LOQ = 0.043), and kaempferol-3-*O*-glucoside (*y* = 17660*x* − 10681; LOD = 0.023, LOQ = 0.072). Standard calibration curves were prepared in a concentration range 0.1–1000 mg/L with five different concentrations and five repetitions for each point. 

For each sample solution, three analyses were carried out and the average of the peak areas was considered; the results are expressed as µg standard equivalents/g fraction (dw) ± SD.

### 4.4. Antioxidant Activity

#### 4.4.1. Free Radical Scavenging Activity

The free radical scavenging activity of *Bff*-EAF was evaluated by the DPPH (2,2-diphenyl-1-picrilidrazil) test, using the method of Ohnishi et al. [45]. The fraction was tested at different concentrations (0.0625–2 mg/mL) using butylated hydroxytoluene (BHT) as the reference standard. A volume of 0.5 mL of each sample solution was mixed with 3 mL of freshly prepared 0.1 mM DPPH methanolic solution; then the samples were incubated in the dark for 20 min at room temperature. After the incubation period, the absorbance of the solutions was measured at λ = 517 nm using an UV–Vis spectrophotometer (model UV-1601, Shimadzu, Milan, Italy). The scavenging activity was measured as the decrease in absorbance of the samples versus DPPH standard solution. The results were obtained from the average of three independent experiments, and are reported as mean radical scavenging activity percentage (%) ± SD and mean 50% inhibitory concentration (IC_50_) ± SD.

#### 4.4.2. Reducing Power

The reducing power of *Bff*-EAF was determined according to the Fe^3+^-Fe^2+^ transformation method reported by Oyaizu [46]. The fraction was tested in the concentration range 0.0625–2 mg/mL and ascorbic acid and BHT were used as reference standards. A volume of 1 mL of each sample was mixed with 2.5 mL of phosphate buffer (0.2 M, pH 6.6) and 2.5 mL of 1% *w*/*v* potassium ferricyanide. After incubation at 50 °C for 20 min and rapid cooling, 2.5 mL of 10% *v*/*v* trichloroacetic acid was added and the mixture was centrifuged for 10 min at 3000 rpm at a temperature of 4 °C; then, 2.5 mL of solution taken from the upper layer was mixed with 2.5 mL of distilled water and 0.5 mL of 0.1% *w*/*v* ferric chloride. The blank was prepared by a similar procedure, replacing the sample solution with an equal volume of solvent. After 10 min of incubation in the dark at room temperature, the absorbances of the samples were measured at λ = 700 nm. Results were obtained from the average of three independent determinations and were expressed as mean absorbance values ± SD and ascorbic acid equivalents (ASE/mL).

#### 4.4.3. Ferrous Ion (Fe^2+^) Chelating Activity

The chelating activity of *Bff*-EAF was estimated by measuring the formation of the Fe^2+^-ferrozine complex, according to the method previously reported by Kumar and colleagues, with slight modifications [47]. The fraction was tested in the concentration range 0.0625–2 mg/mL, using ethylenediaminetetraacetic acid (EDTA) as the reference standard. An aliquot of each sample (1 mL) was mixed with 0.5 mL of distilled water and 50 μL of a 2 mM ferrous chloride solution; subsequently, 0.1 mL of a 5 mM ferrozine solution was added to initiate the reaction. The control was prepared by substituting an equal volume of solvent for the sample solution. The reaction mixture was shaken vigorously and incubated in the dark at room temperature for 10 min; then, the absorbance of the solution was measured spectrophotometrically at λ = 562 nm. The results, obtained from the average of three independent experiments, are reported as mean inhibition of the ferrozine-Fe^2+^ complex formation (%) ± SD and IC_50_ ± SD.

### 4.5. Cytotoxic Activity

#### 4.5.1. Cell Culture and Treatments

CaCo-2 (human colorectal adenocarcinoma cells) and HFF-1 (human normal foreskin fibroblasts) cell lines were obtained by ATCC cell bank, Rockville, MD, USA. CaCo-2 cells were cultured in MEM (Minimum Essential Medium Eagle) containing 10% *v*/*v* fetal bovine serum (FBS), 100 U/mL penicillin, and 100 μg/mL streptomycin, in 5% CO_2_ at 37 °C. HFF-1 cell line was maintained in DMEM (Dulbecco’s Modified Eagle Medium) supplemented with 15% *v*/*v* FBS, 4.5 g/L glucose, 100 U/mL penicillin, and 100 mg/mL streptomycin, in 95% humidified air with 5% CO_2_, at 37 °C. HFF-1 cell line was used as in vitro human model for preliminary toxicity screening. Cells were seeded in 96 well plates for MTT assay and in 6 well plates for LDH release and ROS determinations. At sub-confluent conditions CaCo-2 cells were treated with different concentrations of *Bff*-EAF for 72 h. The fraction was dissolved in medium to obtain the final concentrations ranging from 0.0625 to 1 mg/mL. After the treatments, cells were scraped, washed with PBS and subsequently utilized for the analysis.

#### 4.5.2. MTT Test

Cell viability was measured colorimetrically with tetrazolium salts; this assay evaluates the ability of cells to reduce, by means of mitochondrial succinic dehydrogenase, the bromide of 3- (4,5-dimethylthiazol-2-yl) -2,5-diphenyl tetrazolium (MTT) [48]. The optical density was measured with a microplate spectrophotometer reader (Titertek Multiskan, Flow Laboratories, Helsinki, Finland) at λ = 570 nm. Results are expressed as percentage cell viability respect to control (untreated cells). 

#### 4.5.3. Determination of Lactic Dehydrogenase Release

Lactic dehydrogenase (LDH) release was measured to evaluate the presence of cell necrosis as a result of cell disruption, subsequent to membrane rupture. Enzyme activity was measured spectrophotometrically at λ = 340 nm in the culture medium and in the cellular lysates, by analyzing the β-nicotinamide-adenine dinucleotide (NADH) reduction [49]. The percentage of LDH release was calculated as the percentage of the total amount, considered as the sum of the enzymatic activity present in the cellular lysate and that in the culture medium. 

#### 4.5.4. Determination of Reactive Oxygen Species

CaCo-2 and HFF-1 cells seeded in 6-well plates, untreated and treated for 72 h with *Bff*-EAF at different concentrations (0.0625–1 mg/mL) were incubated for 30 min with the fluorescent probe 2′,7′-dichlorofluorescein diacetate (DCFH-DA) and processed as previously described [50]. After the incubation with the probe, cells were washed with PBS and subsequently exposed to 1 mL of digitonin (2.5 mg/mL) for 1 h at 20 °C, then scraped, and centrifuged at 4 °C, 13.000× *g*, for 10 min. The obtained supernatants were used to determine ROS levels. The fluorescence (corresponding to the oxidized radical species 2′,7′-dichlorofluorescein, DCF) was monitored spectrofluorometrically (excitation, λ = 488 nm; emission, λ = 525 nm). Results are expressed as fluorescence intensity/mg protein and for each sample, the total protein content was determined using BioTek Synergy HTX multimode reader (Agilent, Santa Clara, CA, USA) by measuring the absorbance difference at λ = 280 and λ = 260.

#### 4.5.5. Statistical Analysis

Statistical analyses were performed by one-way and two-way ANOVA. Results were obtained from at least the average of three independent experiments and were expressed as mean ± standard deviation (SD). Data analyses were run with GraphPad Prism v.6 (GraphPad Software, San Diego, CA, USA). For all experiments, * *p* < 0.001 was considered to be significant.

## 5. Conclusions

In a previous work carried out by our research team, the antioxidant properties of the leaf hydroalcoholic extract of *B. fruticulosa* subsp. *fruticulosa* have been demonstrated, and it was hypothesized that the phenolic compounds detected in the extract could be responsible for such antioxidant effects. In this study, the role of the phenolic compounds in the antioxidant properties of the extract was investigated. Based on the results of the in vitro antioxidant tests carried out for the phenolic-rich fraction obtained from *B. fruticulosa* subsp. *fruticulosa* crude extract (*Bff*-EAF), it is possible to state that phenolic compounds contained in the fraction proved to be the main responsible for the primary antioxidant activity highlighted in the crude extract, whereas the good chelating properties of the extract can only partially be traced back to these compounds. Furthermore, *Bff*-EAF exhibited cytotoxic activity against CaCo-2 cells, reducing cell proliferation at all tested concentrations, alongside a redox state destabilization modulated by the antioxidant and pro-oxidant activities exerted at the lower and higher concentrations tested, respectively.

Taken together, these findings show that the leaves of *B. fruticulosa* subsp. *fruticulosa* represent a source of phenolic compounds with antioxidant and antiproliferative activity, indicating this edible species that grows wild in Sicily as a resource of bioactive compounds that could potentially be used as natural agents for health promotion, also providing a substantial contribution to the knowledge of this species hitherto little studied.

## Figures and Tables

**Table 1:** HPLC-PDA/ESI-MS (negative ionization mode) polyphenolic fingerprint of *Bff*-EAF. Results are expressed as µg/g fraction ± SD (*n* = 3).

N.	t_R_ (min)	UV_max_ (nm)	[M-H]^−^	Fragments	Compound	*Bff*-EAF (µg/g)	Refs.
1	29.12	314	325	-	Unknown	Nq	-
2	33.03	328	355	193	Feruloyl-glucose	Nq	[21]
3	35.10	328	385	223	Sinapic acid-glucose	Nq	[21]
4	44.24	322	625	301	Quercetin-*O*-dihexoside	8.77 ± 0.29	[13,21,22]
5	44.84	327	753	341	1,2-Disinapoyl-gentiobiose	Nq	[13,20,23]
6	44.98	332	753	341	1,2-Disinapoyl-gentiobiose isomer	Nq	[13,21,22]
7	46.42	323	723	223, 193	Disinapoyl-feruloyl-triglucoside	Nq	[13,21,22]
8	47.65	321	723	223, 193	Disinapoyl-feruloyl-triglucoside isomer	Nq	[13,21,22]
9	48.02	332	787	301, 353	Quercetin 3-*O*-sophoroside-7-*O*-glucoside	28.00 ± 2.44	[13,21,22]
10	49.55	323	693	387	1,2-Diferuloylgentiobiose	Nq	[13,21,22]
11	49.59	329	423	-	Unknown	Nq	-
12	50.64	327	801	443	Unknown	Nq	-
13	51.59	327	609	285	Kaempferol-3-*O*-diglucoside	12.24 ± 2.02	[13,21,22]
14	53.14	333	963	801	Kaempferol-3-*O*-hydroxyferuloylsophoroside-7-*O*-glucoside	22.47 ± 1.21	[13,21,22]
15	54.22	266, 331	771	285	Kaempferol-3-triglucoside	68.95 ± 1.79	[13,21,22]
16	54.76	317	771	285, 353	Kaempferol-3-*O*-sophoroside-7-*O*-glucoside	12.76 ± 0.48	[13,21,22]
17	55.25	329	977	815, 353	Kaempferol-3-*O*-sinapoylsophoroside-7-*O*-glucoside	18.59 ± 2.06	[13,21,22]
18	55.69	350	463	301	Quercetin 3-*O*-glucoside	11.77 ± 0.27	[13,21,22]
19	59.10	267, 328	785	285	Kaempferol-feruloyldihexoside	50.52 ± 0.53	[13,21,22]
20	60.69	266, 327	857	-	Unknown	Nq	-
21	61.07	267, 317	755	-	Unknown	Nq	-
22	62.02	345	447	285	Kaempferol-3-*O*-glucoside	23.06 ± 0.17	[22]
23	63.7	346	477	285	Isorhamnetin-*O*-glucoside	24.66 ± 1.05	[22]
24	65.31	327	871	-	Unknown	Nq	-
25	74.02	327	959	341	Trisinapoylgentiobiose	Nq	[21]
26	79.39	326	869	527	1,2,2′-Triferuloylgentiobiose	Nq	[24]

Nq: not quantified.

## Data Availability

Not applicable.

## Sample Availability

Samples of the compounds are not available from the authors.

## References

[1] Rahman M.M., Rahaman M.S., Islam M.R., Rahman F., Mithi F.M., Alqahtani T., Almikhlafi M.A., Alghamdi S.Q., Alruwaili A.S., Hossain M.S. (2022). Role of phenolic compounds in human disease: Current knowledge and future prospects. Molecules.

[2] Cory H., Passarelli S., Szeto J., Tamez M., Mattei J. (2018). The role of polyphenols in human health and food systems: A mini-review. Front. Nutr..

[3] Jahangir M., Kim H.K., Choi Y.H., Verpoorte R. (2009). Health-affecting compounds in Brassicaceae. Compr. Rev. Food Sci. Food Saf..

[4] Doniec J., Florkiewicz A., Socha R., Filipiak-Florkiewicz A. (2022). Polyphenolic acid content in *Brassica* vegetables during hydrothermal treatment with salt addition. J. Food Process. Preserv..

[5] Gonçalves E.M., Alegria C., Abreu M., Lang M. (2013). Benefits of *Brassica* Nutraceutical Compounds on Human Health. Brassicaceae—Characterization, Functional Genomics and Health Benefits.

[6] Soengas Fernández M.D.P., Sotelo Pérez T., Velasco Pazos P., Cartea González M.E. (2011). Antioxidant properties of *Brassica* vegetables. Funct. Plant Sci. Biotechnol..

[7] Cartea M.E., Francisco M., Soengas P., Velasco P. (2010). Phenolic compounds in *Brassica* vegetables. Molecules.

[8] Picchi V., Lo Scalzo R., Tava A., Doria F., Argento S., Toscano S., Treccarichi S., Branca F. (2020). Phytochemical characterization and in vitro antioxidant properties of four *Brassica* wild species from Italy. Molecules.

[9] Ramirez D., Abellán-Victorio A., Beretta V., Camargo A., Moreno D.A. (2020). Functional ingredients from Brassicaceae species: Overview and perspectives. Int. J. Mol. Sci..

[10] Favela-González K.M., Hernández-Almanza A.Y., De la Fuente-Salcido N.M. (2020). The value of bioactive compounds of cruciferous vegetables (*Brassica*) as antimicrobials and antioxidants: A review. J. Food Biochem..

[11] Malfa G.A., Acquaviva R., Bucchini A.A.E., Ragusa S., Raimondo F.M., Spadaro V. (2020). The Sicilian wild cabbages as biological resources: Taxonomic update and a review on chemical constituents and biological activities. Flora Medit..

[12] Sánchez-Mata M.C., Cabrera Loera R.D., Morales P., Fernández-Ruiz V., Cámara M., Díez Marqués C., Pardo-de-Santayana M., Tardío J. (2012). Wild vegetables of the Mediterranean area as valuable sources of bioactive compounds. Genet. Resour. Crop. Evol..

[13] Cavò E., Taviano M.F., Davì F., Cacciola F., Oulad El Majdoub Y., Mondello L., Ragusa M., Condurso C., Merlino M., Verzera A. (2022). Phenolic and volatile composition and antioxidant properties of the leaf extract of *Brassica fruticulosa* subsp. *fruticulosa (Brassicaceae)* growing wild in Sicily (Italy). Molecules.

[14] Ficarra P., Scaccabarozzi S. (2019). Brassica fruticulosa. Dalla Natura Alla Tavola. Buoni da Mangiare: Erbe e Frutti Selvatici Delle Vallate dei Nebrodi.

[15] Zarbà C., Allegra V., Zarbà A.S., Zocco G. (2019). Wild leafy plants market survey in Sicily: From local culture to food sustainability. AIMS Agric. Food.

[16] Guarrera P.M. (2009). Le piante nelle tradizioni popolari della Sicilia. Erboristeria Domani.

[17] Pasta S., La Rosa A., Garfì G., Marcenò C., Gristina A.S., Carimi F., Guarino R. (2020). An updated checklist of the sicilian native edible plant: Preserving the traditional ecological knowledge of century-old agro-pastoral landscapes. Front. Plant Sci..

[18] Romano D., Tribulato A., Toscano S., Scuderi D. (2013). Ethnobotanical uses of *Brassicaceae* in Sicily. Acta Hortic.

[19] Lentini F., Venza F. (2007). Wild food plants of popular use in Sicily. J. Ethnobiol. Ethnomed..

[20] Munteanu I.G., Apetrei C. (2021). Analytical methods used in determining antioxidant activity: A review. Int. J. Mol. Sci..

[21] Sun J., Zhenlei X., Long-ze L., Gene E.L., Qin W., James M.H., Pei C. (2013). Profiling polyphenols in five *Brassica* species microgreens by UHPLC-PDA-ESI/HRMS^n^. J. Agric. Food Chem..

[22] Oulad El Majdoub Y., Alibrando F., Cacciola F., Arena K., Pagnotta E., Matteo R., Micalizzi G., Dugo L., Dugo P., Mondello L. (2020). Chemical characterization of three accessions of *Brassica juncea* L. extracts from different plant tissues. Molecules.

[23] Miceli N., Cavò E., Ragusa M., Cacciola F., Mondello L., Dugo L., Acquaviva R., Malfa G.A., Marino A., D’Arrigo M. (2020). *Brassica incana* Ten. (Brassicaceae): Phenolic constituents, antioxidant and cytotoxic properties of the leaf and flowering top extracts. Molecules.

[24] Phenol Explorer. http://phenol-explorer.eu/.

[25] Morales G., Paredes A. (2014). Antioxidant activities of *Lampaya medicinalis* extracts and their main chemical constituents. BMC Compl. Altern. Med..

[26] Alam M.N., Bristi N.J., Rafiquzzaman M. (2013). Review on in vivo and in vitro methods evaluation of antioxidant activity. Saudi Pharm. J..

[27] Malfa G.A., Tomasello B., Acquaviva R., Genovese C., La Mantia A., Cammarata F.P., Ragusa M., Renis M., Di Giacomo C. (2019). *Betula etnensis* Raf. (Betulaceae) extract induced HO-1 expression and ferroptosis cell death in human colon cancer cells. Int. J. Mol. Sci..

[28] Ayoka T.O., Ezema B.O., Eze C.N., Nnadi C.O. (2022). Antioxidants for the prevention and treatment of non-communicable diseases. J. Explor. Res. Pharmacol..

[29] Craft D., Kerrihard A.L., Amarowicz R., Pegg R.B. (2012). Phenol-based antioxidants and the *in vitro* methods used for their assessment. Compr. Rev. Food Sci. Food Saf..

[30] Mladěnka P., Macáková K., Filipský T., Zatloukalová L., Jahodář L., Bovicelli P., Silvestri I.P., Hrdina R., Saso L. (2011). *In vitro* analysis of iron chelating activity of flavonoids. J. Inorg. Biochem..

[31] Han H., Baik B.-K. (2008). Antioxidant activity and phenolic content of lentils (*Lens culinaris*), chickpeas (*Cicer arietinum* L.), peas (*Pisum sativum* L.) and soybeans (*Glycine max*), and their quantitative changes during processing. Int. J. Food Sci. Technol..

[32] Kotha R.R., Tareq F.S., Yildiz E., Luthria D.L. (2022). Oxidative stress and antioxidants—A critical review on in vitro antioxidant assays. Antioxidants.

[33] Salehi B., Quispe C., Butnariu M., Sarac I., Marmouzi I., Kamle M., Tripathi V., Kumar P., Bouyahya A., Capanoglu E. (2021). Phytotherapy and food applications from *Brassica* genus. Phytother. Res..

[34] Avato P., Argentieri M.P. (2015). Brassicaceae: A rich source of health improving phytochemicals. Phytochem. Rev..

[35] Spissu Y., Gil K.A., Dore A., Sanna G., Palmieri G., Sanna A., Cossu M., Belhadj F., Gharbi B., Pinna M.B. (2023). Anti- and pro-oxidant activity of polyphenols extracts of syrah and chardonnay grapevine pomaces on melanoma cancer cells. Antioxidants.

[36] D’Archivio M., Santangelo C., Scazzocchio B., Vari R., Filesi C., Masella R., Giovannini C. (2008). Modulatory effects of polyphenols on apoptosis induction: Relevance for cancer prevention. Int. J. Mol. Sci..

[37] Zhao Y., Zhan J., Wang Y., Wang D. (2022). The relationship between plant-based diet and risk of digestive system cancers: A meta-analysis based on 3,059,009 subjects. Front. Public Health..

[38] Lü J.M., Lin P.H., Yao Q., Chen C. (2010). Chemical and molecular mechanisms of antioxidants: Experimental approaches and model systems. J. Cell. Mol. Med..

[39] León-González A.J., Auger C., Schini-Kerth V.B. (2015). Pro-oxidant activity of polyphenols and its implication on cancer chemoprevention and chemotherapy. Biochem. Pharmacol..

[40] Tang D., Chen X., Kang R., Kroemer G. (2021). Ferroptosis: Molecular mechanisms and health implications. Cell Res..

[41] Gao X., Ohlander M., Jeppsson N., Bjork L., Trajkovski V. (2000). Changes in antioxidant effects and their relationship to phytonutrients in fruit of sea buckthorn (*Hippophae rhamnoides* L.) during maturation. J. Agric. Food Chem..

[42] Arena K., Trovato E., Cacciola F., Spagnuolo L., Pannucci E., Guarnaccia P., Santi L., Dugo P., Mondello L., Dugo L. (2022). Phytochemical Characterization of *Rhus coriaria* L. Extracts by Headspace Solid-Phase Micro Extraction Gas Chromatography, Comprehensive Two-Dimensional Liquid Chromatography, and Antioxidant Activity Evaluation. Molecules.

[43] Asraoui F., Kounnoun A., Cacciola F., El Mansouri F., Kabach I., Oulad El Majdoub Y., Alibrando F., Arena K., Trovato E., Mondello L. (2021). Phytochemical Profile, Antioxidant Capacity, α-Amylase and α-Glucosidase Inhibitory Potential of Wild Moroccan *Inula viscosa* (L.) Aiton Leaves. Molecules.

[44] El Cadi H., El Bouzidi H., Selama G., Ramdan B., Oulad El Majdoub Y., Alibrando F., Arena K., Palma Lovillo M., Brigui J., Mondello L. (2021). Elucidation of Antioxidant Compounds in Moroccan *Chamaerops humilis* L. Fruits by GC–MS and HPLC–MS Techniques. Molecules.

[45] Ohnishi M., Morishita H., Iwahashi H., Shitzuo T., Yoshiaki S., Kimura M., Kido R. (1994). Inhibitory effects of chlorogenic acid on linoleic acid peroxidation and haemolysis. Phytochemistry.

[46] Oyaizu M. (1986). Studies on products of browning reaction: Antioxidative activities of products of browning reaction prepared from glucosamine. Jpn J. Nutr. Diet..

[47] Kumar T.S., Shanmugam S., Palvannan T., Kumar V.M.B. (2008). Evaluation of antioxidant properties of *Elaeocarpus ganitrus* Roxb. leaves. Iran. J. Pharm. Res..

[48] Malfa G.A., De Leo M., Tundis R., Braca A., Loizzo M.R., Di Giacomo C., Raimondo F.M., Bucchini A.E.A., Acquaviva R. (2022). Biological Investigation and Chemical Study of *Brassica villosa* subsp. *drepanensis* (Brassicaeae) Leaves. Molecules.

[49] Acquaviva R., Tomasello B., Di Giacomo C., Santangelo R., La Mantia A., Naletova I., Sarpietro M.G., Castelli F., Malfa G.A. (2021). Protocatechuic Acid, a simple plant secondary metabolite, induced apoptosis by promoting oxidative stress through HO-1 downregulation and p21 upregulation in colon cancer cells. Biomolecules.

[50] Tomasello B., Malfa G.A., La Mantia A., Miceli N., Sferrazzo G., Taviano M.F., Di Giacomo C., Renis M., Acquaviva R. (2021). Anti-adipogenic and anti-oxidant effects of a standardised extract of Moro blood oranges (*Citrus sinensis* (L.) Osbeck) during adipocyte differentiation of 3T3-L1 preadipocytes. Nat. Prod. Res..

